# Developmental Toxicity of Diethylnitrosamine in Zebrafish Embryos/Juveniles Related to Excessive Oxidative Stress

**DOI:** 10.1007/s11270-018-3739-8

**Published:** 2018-02-22

**Authors:** Danping Huang, Hanmin Li, Qidi He, Weiqu Yuan, Zuanguang Chen, Hongzhi Yang

**Affiliations:** 1 Guangdong Key Laboratory of Liver Disease Research, The Third Affiliated Hospital, Sun Yat-sen University, Tianhe Road No. 600, Guangzhou, 510000 China; 2 Hubei Provincial Hospital of Traditional Chinese Medicine, Garden Hill No. 4 Wuchang District, Wuhan, 430061 China; 3 Hubei Province Academy of Traditional Chinese Medicine, No.856 Luoyu Road, Hongshan District, Wuhan, 430074 China; 4 School of Pharmaceutical Sciences, Sun Yat-sen University, Guangzhou, 510006 China; 5 The fourth Clinical Medical College of Guangzhou University Chinese Med, Shen zhen, 518000 China

**Keywords:** Developmental toxicity, Oxidative stress, Diethylnitrosamine, Teratogenicity, Zebrafish

## Abstract

Diethylnitrosamine (DEN) is present in food, water, and daily supplies and is regarded as a toxicant of carcinogenicity. The developmental toxicity of DEN has been rarely reported as yet. In this study, zebrafish were exposed to different concentrations of DEN at 6 h post-fertilization (hpf) to access embryonic toxicity of the compound. The results show that DEN resulted in negative effects of hatching rate, heartbeat, body length, and spontaneous movement. Deformities, including notochord malformation, pericardium edema, embryonic membrane turbidity, tail hypoplasia, yolk sac deformity, and growth retardation, happened during exposure period. Moreover, production of reactive oxygen species (ROS) significantly increased after DEN treatment. Then, alterations of the expression level of oxidative stress-related genes were observed in our results. To our knowledge, this is the first study concerning the effect of DEN on zebrafish. And from the information of our research, we speculated that development toxicity of DEN should be related to the excessive oxidative stress.

## Introduction

*N*-nitrosamines (NAs) are well known for its strong carcinogenicity. Stable nitrosamines are mainly synthesized from secondary amines which is generated from pesticides, herbicide, and nitrogen fertilizers (Park et al. 2015). Researchers paid close attention to *N*-nitrosamines’ carcinogenicity which was confirmed to cause a series of digestive tract cancers such as esophageal carcinoma, gastric cancer, hepatic carcinoma, pancreatic adenocarcinoma, and so on (Loeppky and Goelzer 2002; Park et al. 2015). Technology for accurate quantification of NAs in various stuffs is also highly concerned (Chen et al. 2014; Li et al. 2014a, b). However, developmental toxicity of NAs in juvenile period is rarely documented. Nowadays, the effect of wide application of pesticides, herbicides, and nitrogen fertilizers in agriculture causes excessive production of NAs, which inevitably threatens the aquatic life. Therefore, it is necessary to evaluate related toxicity of NAs in the process of development.

Diethylnitrosamine (DEN), the usual form of NAs, is one of *n*-nitrosamines in the agricultural food products, seafood, and edible oil water et al (Park et al. 2015). The US Environmental Protection Agency (EPA) has set the maximum limitation concentration of it in water (Crews 2010). And DEN is frequently used for making a series of cancer models in laboratory (Y.-J. Chen et al. 2016; Jayaprakash et al. 2015; Pacheco-Rivera et al. 2016). Hence, DEN was conducted in this study as a representative of NAs.

Zebrafish (*Danio rerio*), a promising model organism, possesses the nature of small size, short lifecycle, high reproducibility, and transparent embryo (Dai et al. 2014; Yang et al. 2016; Yang et al. 2011). Additionally, it is economic and easy to husbandry. More remarkable, zebrafish share many similar characteristics and conserved mechanisms with human beings (Zhu et al. 2015; Chen and Cheng 2014). Therefore, scientists have established liver and pancreas cancer models in zebrafish by long-time DEN interference (Mizgireuv and Revskoy 2006; Mizgirev and Revskoy 2010) for fundamental research. Moreover, observation of its development has been applied to the access of teratogenic toxicants (Antkiewicz et al. 2005; Reimers et al. 2004; Chen and Cheng 2014).

In the present study, zebrafish were exposed to different concentrations of DEN and physiological parameters including hatching rate, mortality, abnormality rate, body length, heart rate, and spontaneous movement were observed. The reactive oxygen species (ROS) production were also measured. Finally, genes encoding antioxidant proteins including superoxide dismutase (SOD), catalase (CAT) as well as glutathione peroxidase (GPx) were examined and analyzed. In conclusion, our study aims to provide new insights into the developmental toxicity of DEN.

## Materials and Methods

### Materials

#### Reagents

*N*-nitrosodiethylamine (DEN. CAS number 55–18-5; molecular formula: C_4_H_10_N_2_O; molecular weight 102.14; purity 98.0%), sodium chloride, potassium chloride, calcium chloride, and magnesium sulfate anhydrous were purchased from Aladdin, Shanghai. Tricaine (CAS number 886-86-2; molecular formula C_9_H_11_NO_2_·CH_4_O_3_S; molecular weight 261.29; purity > 97.0%) were purchased from TCI, Shanghai.

#### Animals

AB strain zebrafish embryos were obtained from the zebrafish laboratory of Sun Yat-sen University. All protocols were in accordance with the National Institutes of Health guide for the care and use of laboratory animals (NIH Publications No. 8023, revised 1978) and got the permission of the ethic committee on the Care and Use of Laboratory Animals of Sun Yat-sen University, Guangzhou, China.

### Procedure

#### Maintenance of Zebrafish Embryos

The 6 hpf zebrafish embryos on a piece of gauze were washed by E3 medium to clean the impurities (E3 medium which includes 5 mmol/l NaCl, 0.17 mmol/l KCl, 0.16 mmol/l MgSO_4_, and 0.40 mmol/l CaCl_2_ was prepared by redistilled water, then inflated for half an hour with an air pump to insure enough air for embryos development). Then, zebrafish embryos were put into a culture dish. Filtration of healthy fertilization embryos was arranged under the stereomicroscope (SMZ-T4, Optec, Chongqing, China). After filtration, they were maintained in the 24-well plate with a final volume of 2 ml every well. Finally, we hold them into a thermostat with a standard condition (T = 26 ± 1 °C, 14 h/10 h light/dark cycles).

#### DEN Exposure

We exposed zebrafish embryos to a series of concentrations (50, 100 , 150, and 200 μg/ml). DEN was prepared at a concentration of 1 mg/ml as the stock solution. Stock solution was prepared in E3 medium and then stored at 4 °C. Control group was exposed to E3 fluid. Each group was measured 60 embryos with three parallel treatments. Healthy embryos at 6 hpf were transferred into test solutions in 24-well plates, and each well contained 2 ml of exposure solution. One third of the solution was replaced every day to clean the metabolites.

#### Lethality, Hatchability, and Teratogenicity

Lethality, hatchability, and teratogenicity were calculated at 24, 48, and 72 hpf to study the effect to the life and development toxicity of zebrafish under the stereomicroscope. Sign of death is the absence of heartbeat under microscopy. When larva’s head or tail breaks out of the embryo membrane, hatching is successful. Concrete teratogenic pictures at every stage were taken by the microscopic image acquisition system (OPTEC, Chongqing, China). Counting formulas of lethality, hatchability and teratogenicity at each time point were shown below:

- Lethality (%) = death number/total exposed number × 100%
- Hatch ratio (%) = hatched number/total survival embryos number × 100%
- Abnormality rate (%) = abnormal number/total survival number × 100%
- Dead embryos or larvae were cleaned at each time stage of observation

#### Body Length of Larvae

At 72 hpf, we randomly selected ten normal larvae from each group and placed them upon a glass slide, respectively. Under the anesthetic action of tricaine, larva was adjusted in the position of the abdomen down with a small soft brush. Then, body length was measured using microscopic image acquisition system according to the method as described (Li et al. 2014a, b).

#### Heart Rate of Larvae

Heart of zebrafish is anterior to yolk sac and posterior to the jaw. Heart rate of normal zebrafish is easy to be recognized with a character of palmus. Heart rate was counted for 1 min directly at 72 hpf in 24-well plate under the stereomicroscope vision at the surrounding of appropriate temperature (T = 26 ± 1 °C). Ten larvae in each group were chosen to record heart rate three times, and then the average was calculated.

#### Spontaneous Movement

Spontaneous movement is the muscle contraction featured with tail swing at the embryo period. Data collection was implemented from 23 to 30 hpf. For precise calculation, we recorded the total spontaneous movements of all zebrafish in every well under the views of stereomicroscope. At each time point, we counted for 1 min and repeated three times by two researchers, then made a conversion into the average.

#### ROS Measurement

ROS content of zebrafish after DEN exposure was detected by dichlorofluorescein-diacetate (DCFH-DA) as described by Zhu et al. (2015). At 72 hpf, 10 larvae of each treated group were washed by cold PBS (pH 7.4) twice. Cold buffer (1 mM of MgCl_2_, 0.5 mM of phenylmethyl sulfonylfluoride (PMSF), 0.32 mM of sucrose, and 20 mM of HEPES, pH 7.4) was prepared for tissue homogenizing. Then, the homogenate was centrifuged(15,000×*g*, 4 °C, 20 min) and 20 μl supernatant was added to a 96-well plate and incubated at 27 °C for 5 min. After that, we added DCFH-DA stock solution (8.3 μl, dissolved in DMSO, 10 mg/ml) and PBS (100 μl, pH 7.4) to each well and incubated the plate at 37 °C for 30 min. Measurement of the fluorescence intensity was performed by a microplate reader (Molecular devices, FlexStation 3, American) with excitation and emission at 485 and 530 nm, respectively.

#### Gene Expression Analysis

After DEN-exposed treatment, larvae with normal morphology were washed by ice-cold PBS twice. Total RNA of 15 larvae from each group was extracted at 76 hpf using Trizol reagent. The reverse transcription was performed using 1 μg total RNA by the PrimeScript® RT reagent Kit (TakaRa, Shiga, Japan). Quantitative real-time PCR was run on LightCyclerH 480 II (Roche, Applied Science) using validated primers (Table 1) and SYBR Premix ExTaq II (Takara, Japan) in triplicate. Finally, target gene expression was normalized to housekeeping gene *gapdh* and the transcription level was demonstrated by forms of fold changes.

**Table 1:** Primer sequences of target genes

Gene symbol	Primer sequence	Reference	Accession number
*sod*	F-GTCGTCTGGCTTGTGGAGTG	Jin et al. (2010)	Y12236
	R-TGTCAGCGGGCTAGTGCTT		
*cat*	F-AGGGCAACTGGGATCTTACA	Jin et al. (2010)	AF170069
	R-TTTATGGGACCAGACCTTGG		
*gpx*	F-AGATGTCATTCCTGCACACG	Jin et al. (2010)	AW232474
	R-AAGGAGAAGCTTCCTCAGCC		
*gapdh*	F-GTGGAGTCTACTGGTGTCTTC	DiMuccio et al. (2005)	BC083506
	R-GTGCAGGAGGCATTGCTTACA		

#### Statistics

Data was collected and analyzed by SPSS 20.0 software through one-way ANOVA after checking the normality and homogeneity of variances. Then, post hoc least significant difference (L-S-D) multiple comparison test was conducted. All the data were recorded with forms of mean ± SD. *p* < 0.05 means significant difference.

## Results

### Lethality

**Fig. 1:**
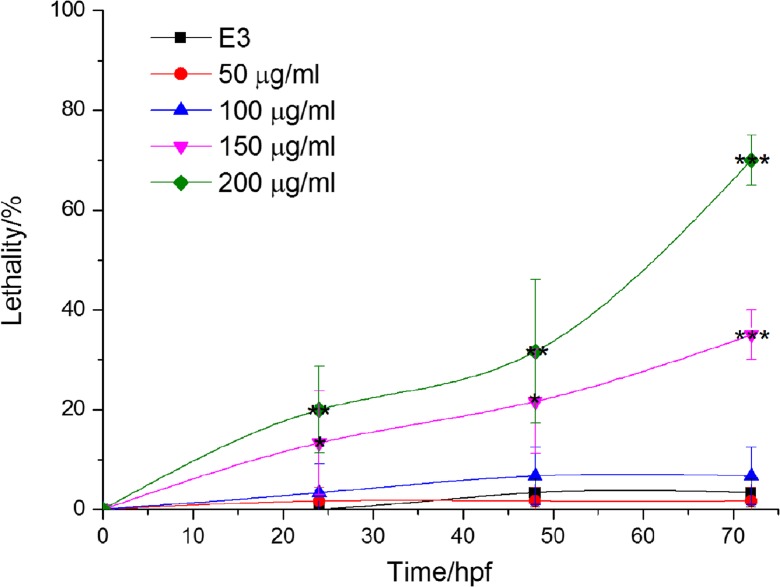
Lethality data of zebrafish at different DEN-exposed time. The asterisks indicate significant difference from the control group at the same time point (*p < 0.05; **p < 0.01; ***p < 0.001). Error bars represent the standard deviation

DEN can lead to death at our first step to assess the risk of DEN on zebrafish. Lethality of zebrafish embryos or larvae at three time points (24, 48, and 72 hpf) was detected and shown in Fig. 1. A concentration-dependent manner can be found in the mortality caused by DEN. Compared with control group, no significant difference existed in the DEN concentration group of 100 μg/ml and below. But what is certainly clear is that notable difference was observed at three time points at concentrations of 150 and 200 μg/ml.

### Hatchability

**Fig. 2:**
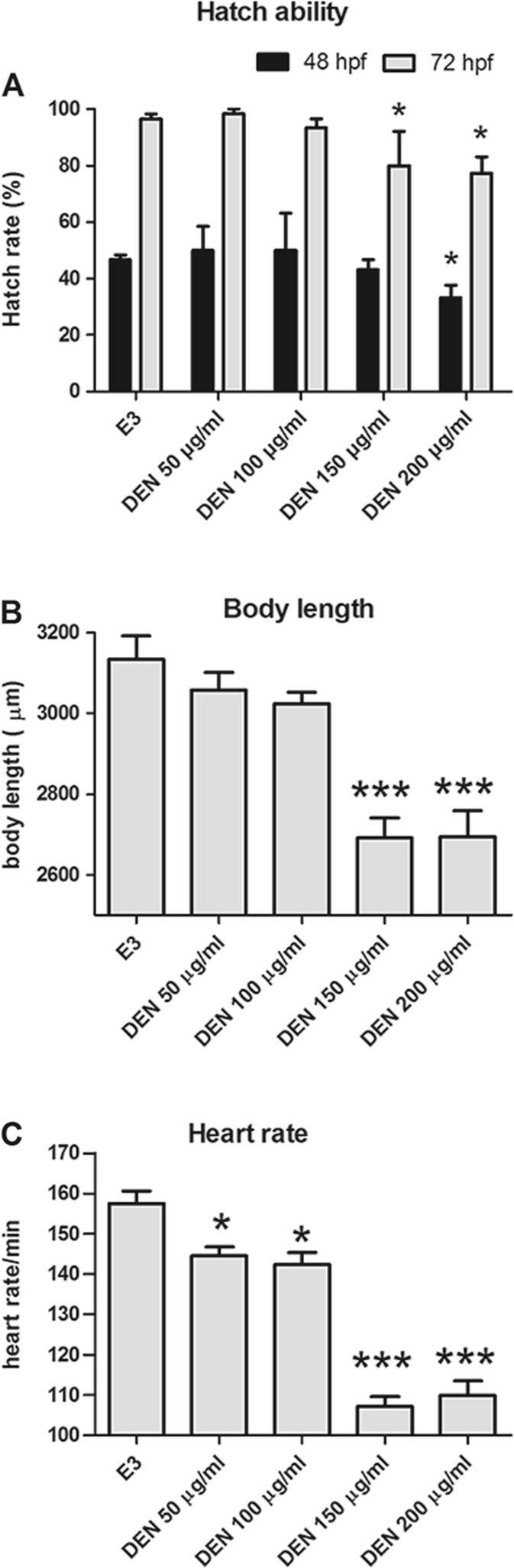
**a** Hatch rate at 48 and 72 hpf after DEN exposure. **b** Body length of zebrafish larvae at 72 hpf after DEN exposure. **c** Heart rate of zebrafish at 72 hpf after DEN treatment. *p < 0.05, ***p < 0.001, significantly different when compared to the control group at the same time point

Zebrafish embryos begin to hatch at 48 hpf and finish hatching at 72 hpf in normal condition. Hatch rate between control and low concentration (50 and 100 μg/ml) ones had no obvious difference. It reduced in the higher groups (150 and 200 μg/ml) (Fig. 2a). At 48 hpf, hatching rate was significantly reduced in the DEN concentrations of 200 μg/ml. At 72 hpf, hatching inhibition was observed when the embryos were exposed to DEN concentrations of 150 and 200 μg/ml.

### Teratogenicity

**Fig. 3:**
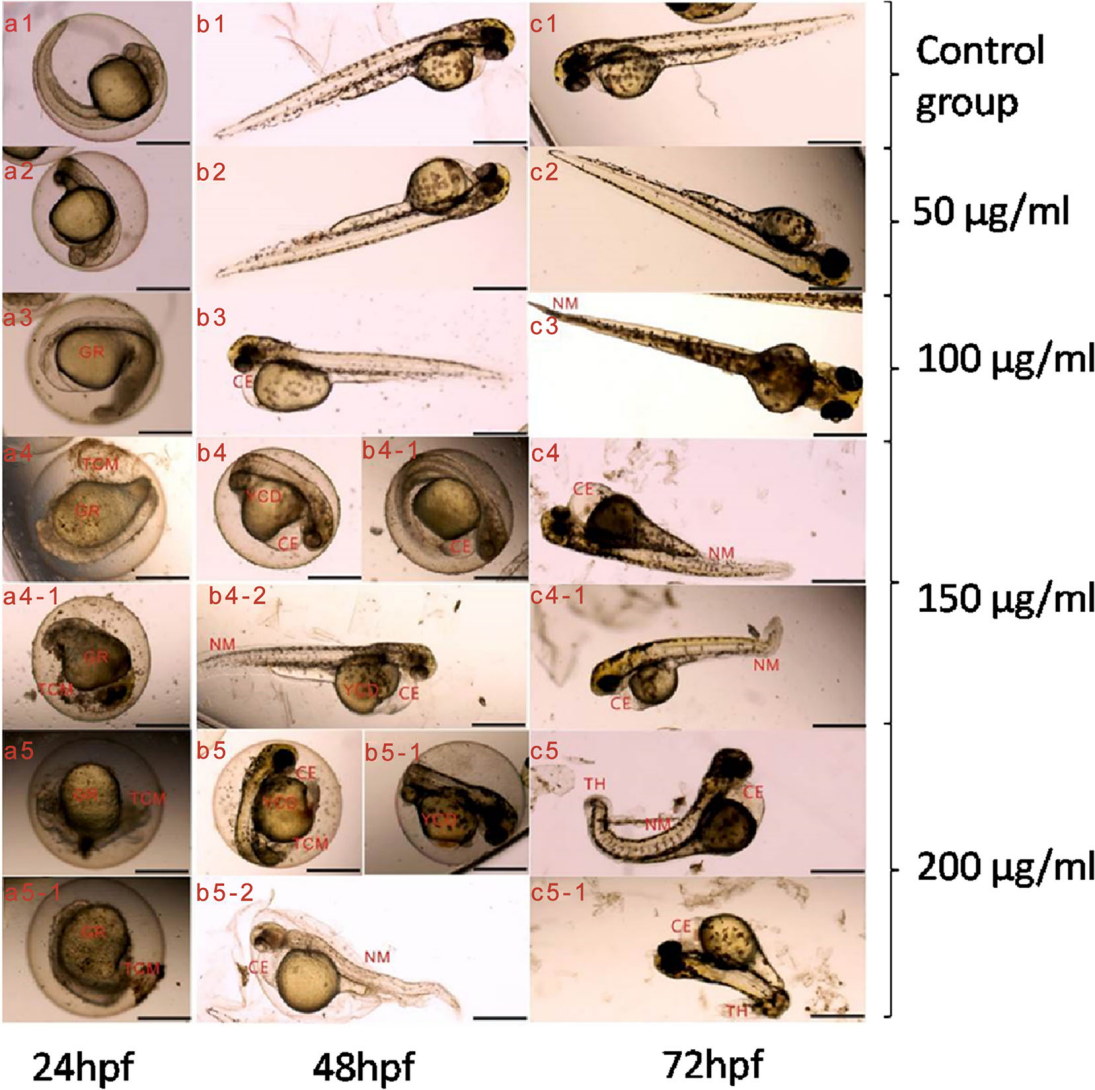
Representative malformation images of zebrafish for different time points (24, 48, 72 hpf). Letter represents different time point (**a** 24 hpf; **b** 48 hpf; **c** 72 hpf). Number represents different group (1 control group; 2 50 μg/ml of DEN; 3 100 μg/ml of DEN; 4 150 μg/ml of DEN; 5: 200 μg/ml of DEN). There were hatched larvae and unhatched embryos existing simultaneously at 48hpf. TCM turbid embryonic membrane, CE cardiac edema, NM notochord malformation, TH tail hypoplasia, YCD Yolk sac deformity, GR growth retardation

A series of morphological deformations were observed under the stimulation of DEN, including spinal curvature, pericardium edema, embryonic membrane turbidity, tail hypoplasia, yolk sac deformity, and growth retardation. Teratogenic ratio increased in a dose-dependent manner (Fig. 3 and Table 2). Compared with the control group, malformations appeared earlier in groups of 150 and 200 μg/ml. In addition, the most prominent malformations were spinal curvature and pericardium edema among these morphological alterations and the phenomenon of pericardium swelling always showed up earlier.

**Table 2:** Teratogenic data of zebrafish exposed to DEN at 72 hpf

Diethylnitrosamine concentration (μg/ml)		0			50			100			150		200			
Parallel classes		1	2	3	1	2	3	1	2	3	1	2	3	1	2	3
Malformations	Notochord malformation	0	0	0	0	0	0	1	0	1	6	6	7	3	2	3
	cardiac edema	0	0	1	1	0	0	1	1	2	6	5	8	3	2	3
	Turbid embryonic membrane	0	0	0	0	1	0	0	0	1	1	0	1	2	2	2
	tail hypoplasia	0	0	0	0	0	0	0	0	0	0	1	0	2	1	0
	Yolk sac deformity	0	0	0	0	0	0	0	0	1	3	3	2	3	1	2
	Growth retardation	0	0	0	0	0	0	0	0	0	1	1	0	1	1	1
Teratogenic embryos		0	0	1	1	1	0	2	1	4	7	6	11	5	5	6
∑teratogenic embryos		1			2			7			24			16		
Malformation %		1.75 ± 3.04			3.42 ± 2.30			12.78 ± 8.73			60.81 ± 15.51***			89.68 ± 9.01***		

Malformation data were presented as mean ± SD (three repeated experiments)

***p < 0.001, compared to the E3 control group

### Body Lengths of Larvae

Apparently, negative correlation existed between body length and concentrations. Body length of zebrafish had no significant difference in the DEN groups of 50 and 100 μg/ml. However, significant inhibition was observed at concentrations of 150 and 200 μg/ml through comparison to the control group (Fig. 2b).

### Heart Rates of Larvae

Similarly, heart rate of larvae had a negative relationship to DEN level. Normal heartbeat of larvae is around 155 times/min. All the exposure groups exhibited slower heart plumps, and the groups of 150 and 200 μg/ml showed significantly abnormal heart rates of approximately 110 times/min (Fig. 2c).

### Spontaneous Movements

**Fig. 4:**
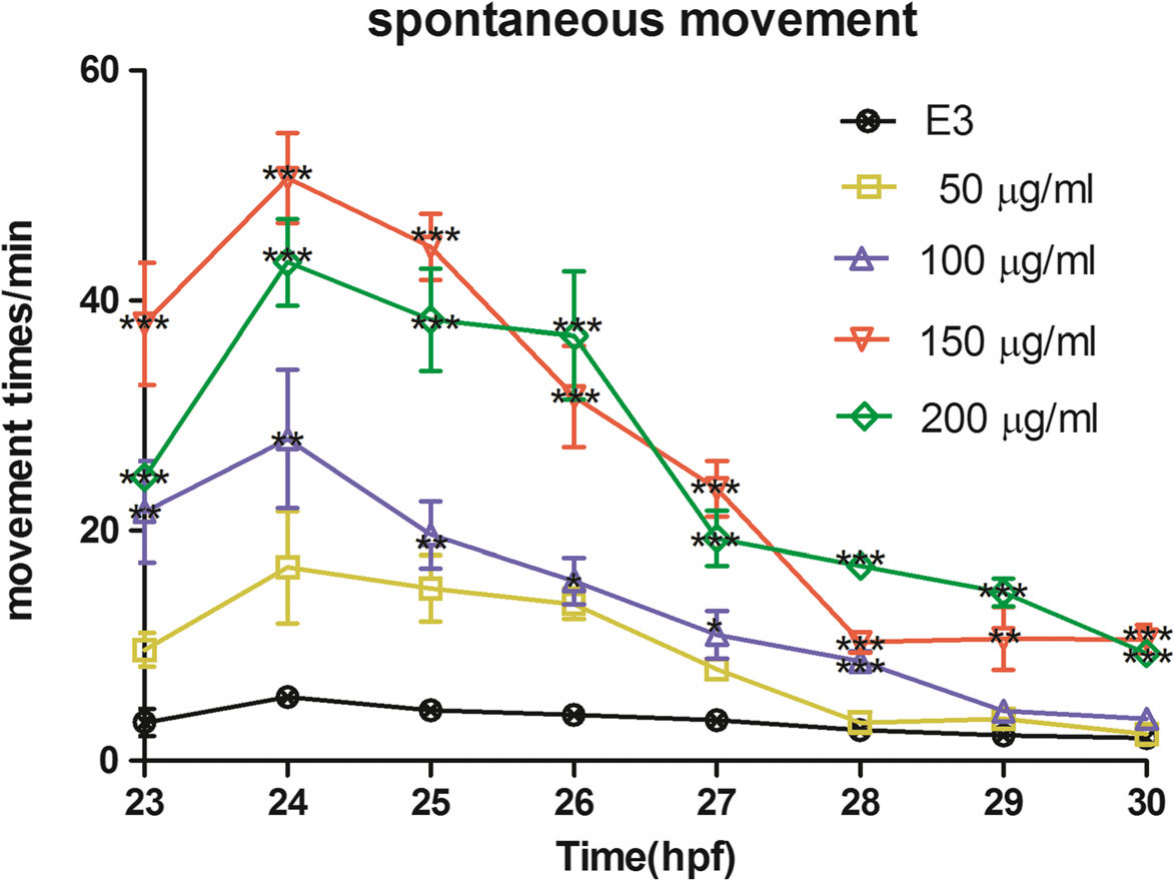
Spontaneous movement of embryos for 1 min from 23 to 30 hpf. Each time point was presented as mean ± SD from three parallel classes. *p < 0.05; **p < 0.01; ***p < 0.001 were all compared to the control group at the same time point

Spontaneous movement alteration is demonstrated in Fig. 4. In time period of 24–30 hpf, the spontaneous movements of embryos continued to decrease over time. Compared to the control group, a distinct rise in frequency could be observed at each time point in the DEN treating groups of 150 and 200 μg/ml. We also observed that significant alteration of spontaneous movement was existing in group of 100 μg/ml from 23 to 28hpf.

### ROS Measurement

**Fig. 5:**
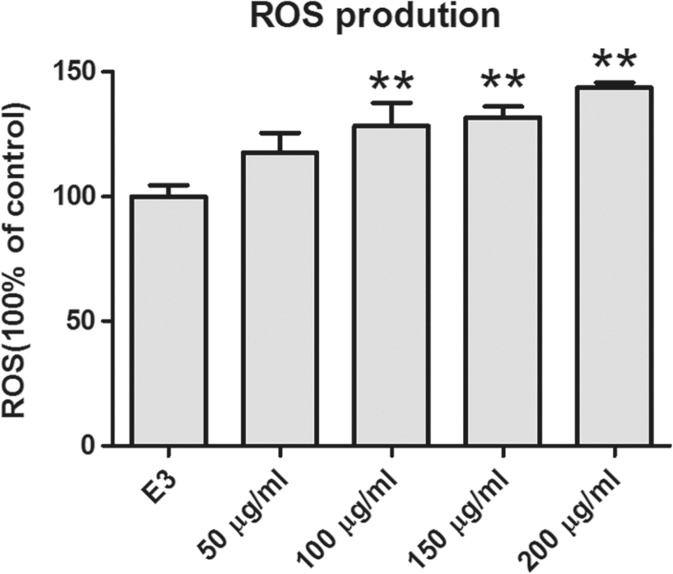
ROS level in different concentrations of DEN-treated embryos at 72 hpf. The asterisks indicate significant differences from the control group (**p < 0.01). Error bars represent the standard deviation

The ROS level of zebrafish after DEN exposure is shown in Fig. 5. ROS content was significantly increased in the 100 μg/ml and higher groups. There is no significant change in ROS concentration in group of 50 μg/ml when compared to the control one.

### Gene Expressions of Anti-Oxidative Stress Genes

**Fig. 6:**
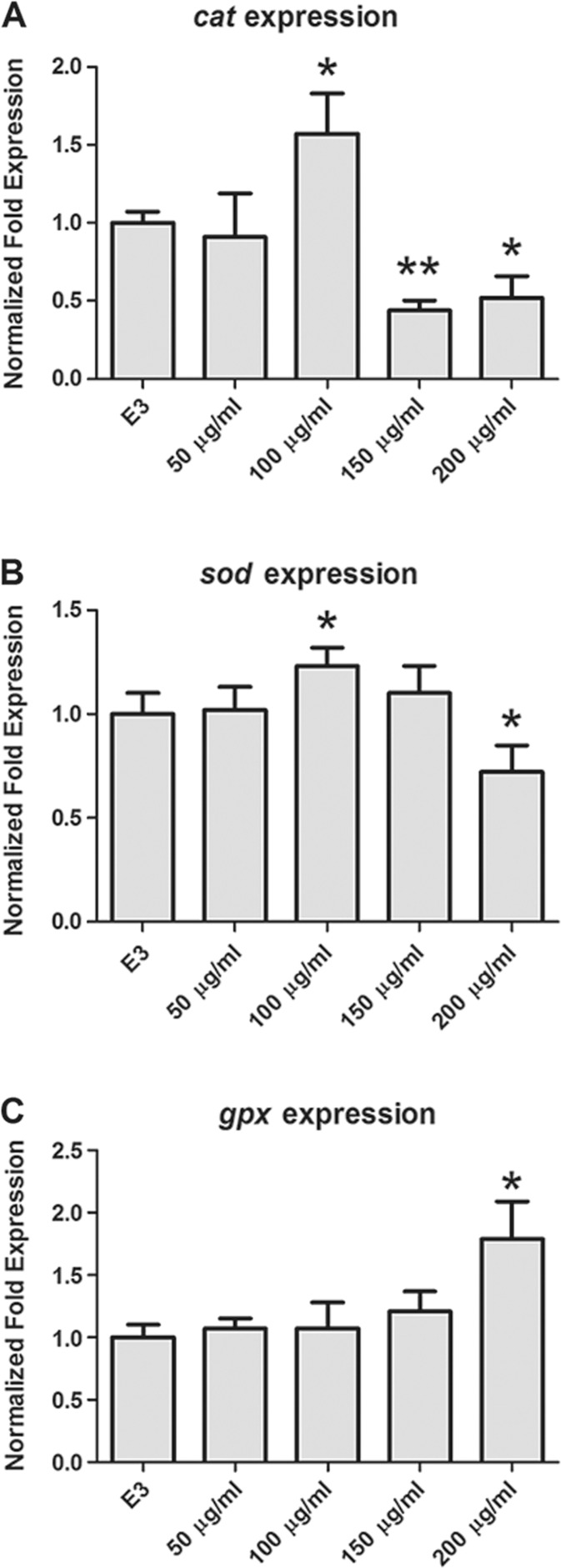
Gene expression after DEN exposure. **a**, **b**, **c** mRNA expression level of anti-oxidative enzyme genes: *cat*, *sod*, *gpx*. *p < 0.05 and **p < 0.01 were all compared to the control group

Here, we detected the mRNA expression level of three genes being involved in oxidative stress response (Fig. 6). Compared with the control group, transcription level of gene *cat* and *sod* increased significantly in group of 100 μg/ml and decreased gradually in groups of higher concentrations (Fig. 6a, b). *gpx* expression quantity only had a significant increase in the highest concentration (200 μg/ml) and showed no obvious variation in other groups (Fig. 6c).

## Discussion

This study shows that DEN had negative effects on the development of zebrafish. We observed that DEN caused adverse impact on hatching rate, heartbeat, body length, and spontaneous movement. Varying degrees of mortality and malformations at different body parts, including yolk sac, pericardium, tail, notochord, and embryonic membrane were also observed. According to the data presented, ROS is over produced in treating groups (100, 150, and 200 μg/ml). It also clearly indicated that the expression of gene-related antioxidant proteins was significantly changed, suggesting the vital role of oxidative stress in the development toxicity of DEN.

In our study, lethality and malformation increased from 48 to 72 hpf, suggesting there is a sensitive time period in response to DEN-caused toxicity. This phenomenon also happened in researches in which zebrafish were exposed to other toxicants. But they performed a distinct time period of lethality and malformation (Reimers et al. 2004; Weigt et al. 2012) which is possibly related to different degrees of potency and metabolism. Variant malformations occurred after exposure to DEN such as notochord malformation, pericardium edema, embryonic membrane turbidity, tail hypoplasia, yolk sac deformity, and growth retardation. Most of similar toxic effects were confirmed by other researchers who applied toxicant such as PBDE 47 (Lema et al. 2007), TCDD (Antkiewicz et al. 2005; Yamauchi et al. 2006), and copper (Johnson et al. 2007) to zebrafish. It is worth noting that morphological deformations mainly occurred in heart. And pericardial edema appeared in earlier time. Inhibition of heart rate was also observed in our study. Therefore, we speculate that development and function of heart can be a potential target of this poisonous substance. The same results were recorded in zebrafish exposed to toxicants such as PFOS (Shi et al. 2008) and cyhalofop-butyl (Zhu et al. 2015), and apoptosis is clarified to be involved in this process according to the existing research, which needs further study for the knowledge of exact mechanism.

Oxidative stress has gained wide attention in contaminant-stimulated toxicity of aquatic organism (Livingstone 2001). It results from the unbalance among antioxidant system, the production of oxygen-free radicals, and other ROS (Valavanidis et al. 2006; Tellez-Banuelos et al. 2009; Paskova et al. 2011). Excess ROS generation was detected in our study, which reminds us of the relationship between oxidative stress and developmental toxicity of DEN. On the other hand, DEN was extensively applied in inducing liver cancer in rats and some transgenic zebrafish (Jayaprakash et al. 2015). Metabolism of DEN is activated by cytochrome P450 enzymes and results in excess oxidative stress, which is involved in the carcinogenicity process. Therefore, the elevated oxidative stress may also contribute to the developmental toxicity induced by DEN.

Scientists have identified lots of genes related to the response of oxidative stress in zebrafish (Craig et al. 2007; Liu et al. 2008). Antioxidant enzymes can response to oxidative stress and assess the elevated levels of ROS. Thus, in plenty of researches, they are considered as the molecular biomarkers of internal antioxidant status and have been applied broadly in the assessment of environmental toxicity of pollutants (Livingstone 2001; Valavanidis et al. 2006; Tilton et al. 2008; Maria et al. 2009; Zhang et al. 2009). Therefore, the representative genes of these proteins (such as SOD, CAT, and GPx) were used to study whether they can serve as endpoints for DEN exposure in zebrafish. SOD gathers in mitochondria and cytosol converts the superoxide anion radical to water and hydrogen peroxide. Then, CAT catalyzes the detoxification of hydrogen peroxide in peroxisomes. They compose the first barrier to combat the ROS damage. GPX is a protein that removes hydrogen peroxide in the nucleus, mitochondria, and cytosol. Its function is similar to CAT but needs the consumption of glutathione. Moreover, CAT and SOD are more sensitive and effective than GPx (Wang et al. 2002; Pandey et al. 2003; Liu et al. 2008; Paskova et al. 2011).

In our study, *sod* and *cat* gene expression level increased significantly in the low concentration group (100 μg/ml) while *gpx* level only increased in highest concentration ones (200 μg/ml). Oxidative stress is in a concentration-dependent manner in our study according to ROS level detected in trail. The result confirmed that response ability of s*od* and *cat* is more sensitively than *gpx* in DEN-caused oxidative stress. Surprisingly, transcription of *sod* and *cat* performed a downtrend in higher concentration groups (150 and 200 μg/ml). It suggested that the oxidative stress with which zebrafish faced is not parallel to the mRNA expression of antioxidant enzymes. Similar phenomenon can also be observed when scientists conducted atrazine to zebrafish. After atrazine exposure, expression level of *cat* and *sod* increased gradually in the lower concentration group but dropped to normal in higher ones (Jin et al. 2010). What is more, it was also observed that other toxicants, including 1-methyl-3-hexylimidazolium bromide (C. Zhang et al. 2017a), pyraclostrobin (Zhang et al. 2017b) enhanced the ROS generation but inhibited the activity of anti-oxidative enzymes in zebrafish. However, there are some researches that indicated that toxicants such as cyhalofop-butyl and heavy metal (cadmium (Cd) and lead (Pb)) promoted the ROS production and increased the mRNA expression of genes related to oxidative stress (SOD, CAT, GPx) (Yin et al. 2017; Zhu et al. 2015). We may speculate that the increased expression of antioxidant enzymes is transient under less oxidative stress. When toxicants’ concentration increased, lower transcription can be measured in antioxidant system (Valavanidis et al. 2006). There are a few explanations that mRNA levels are possibly a snapshot response of the cell activity at any given time, and the anti-oxidative activity might be influenced at the post-translational level of enzymes (Jin et al. 2010). Furthermore, oxidative stress and mRNA level of *sod* and *cat* is not parallel in the liver of smoltifying salmon after exposure to high level of O_2_ according to the research of Olsvik et al. (2005). In a related study of Nile tilapia from a polluted lake, *gpx* activity was lower in liver than in kidney and gill (Pandey et al. 2003; Jin et al. 2008). As we know, liver detoxifies the accumulated toxicants biologically and suffers stronger oxidative stress than other organs. Thus, plenty of exceeded oxidative stress resulted in lower *gpx* transcription. But *gpx* activity only increased in highest concentration group in our study. It might be because *gpx* is less sensitive than *sod* and *cat* under the stimulation of equal oxidative stress. Based on the above, we speculate that antioxidant enzymes’ expression level may decrease when oxidative stress reaches a certain range in which organism could not neutralize it. Exceeded ROS increases transcription of oxidative enzymes primarily, then inhibits, even destroys, one or more links among the processes with the increasing of oxidative stress. Finally, it results in lower expression of antioxidant enzymes. There is no exact explanation for the different responses of anti-oxidative stress system to different compounds in zebrafish. Maybe it depends on variant toxic level, multiple exposure times, different metabolism, and so on. But deeper mechanism is unclear now, which needs to be further studied in more detailed experiments.

## Conclusion

In summary, DEN is hazardous to the development of zebrafish embryos and juveniles. It caused negative effects on several developmental parameters such as hatching rate, heart rate, body length, and spontaneous movement. It induced malformations in various body regions such as yolk sac, pericardium, tail, notochord, and embryonic membrane. The ROS level was affected and gene expressions of antioxidant enzymes were changed after DEN exposure. In conclusion, developmental toxicity of DEN are associated with the excessive oxidative stress in a certain extent. Our results primarily offer detail documents of DEN with respect of development toxicity in zebrafish. It emphasizes the importance of supervisory control in DEN content during the manufacturing operation of daily stuff and rational application of pesticides, herbicide, and so on. Our study can also serve as an animal model to screen toxicides of DEN and anti-oxidative stress medicines.
